# Immune-Escape Mutations Are Prevalent among Patients with a Coexistence of HBsAg and Anti-HBs in a Tertiary Liver Center in the United States

**DOI:** 10.3390/v16050713

**Published:** 2024-04-30

**Authors:** Mukarram Jamat Ali, Pir Ahmed Shah, Khalil Ur Rehman, Satinder Kaur, Vera Holzmayer, Gavin A. Cloherty, Mary C. Kuhns, Daryl T. Y. Lau

**Affiliations:** 1 Liver Center, Division of Gastroenterology and Hepatology, Department of Medicine, Beth Israel Deaconess Medical Center, Harvard Medical School, Boston, MA 02215, USA; mukarram4615@gmail.com (M.J.A.); pirashah2020@gmail.com (P.A.S.); khalilurrehmantajik@gmail.com (K.U.R.); satinder5@hotmail.com (S.K.); 2 Howard University Hospital, Howard University College of Medicine, Washington, DC 20060, USA; 3 Abbott Diagnostics Division, Abbott Laboratories, Abbott Park, IL 60064, USA; vera.holzmayer@abbott.com (V.H.); gavin.cloherty@abbott.com (G.A.C.); mary.kuhns@att.net (M.C.K.)

**Keywords:** HBsAg mutation, vaccine-escape mutant, S gene, ‘α’ determinant region

## Abstract

The concurrent seropositivity of HBsAg and anti-HBs has been described among patients with chronic hepatitis B (CHB), but its prevalence is variable. HBV S-gene mutations can affect the antigenicity of HBsAg. Patients with mutations in the ‘α’ determinant region of the S gene can develop severe HBV reactivation under immunosuppression. In this study at a tertiary liver center in the United States, we evaluated the frequency and virological characteristics of the HBsAg mutations among CHB patients with the presence of both HBsAg and anti-HBs. In this cohort, 45 (2.1%) of 2178 patients were identified to have a coexistence of HBsAg and anti-HBs, and 24 had available sera for the genome analysis of the Pre-S1, Pre-S2, and S regions. The frequency of mutations in the S gene was significantly higher among those older than 50 years (mean 8.5 vs. 5.4 mutations per subject, *p* = 0.03). Twelve patients (50%) had mutations in the ‘α‘ determinant region of the S gene. Mutations at amino acid position 126 were most common in eight subjects. Three had a mutation at position 133. Only one patient had a mutation at position 145—the classic vaccine-escape mutation. Despite the universal HBV vaccination program, the vaccine-escape mutant is rare in our cohort of predominantly Asian patients.

## 1. Introduction

Hepatitis B virus (HBV) remains a significant public health problem despite the availability of effective vaccines. It is a major cause of chronic liver disease, cirrhosis, and hepatocellular carcinoma (HCC). WHO estimated that 296 million people had chronic HBV infection globally [1]. There are HBV genotypes from A to J in different geographical regions, with approximately 8% nucleotide variation in their genomes [2].

**Figure 1:**
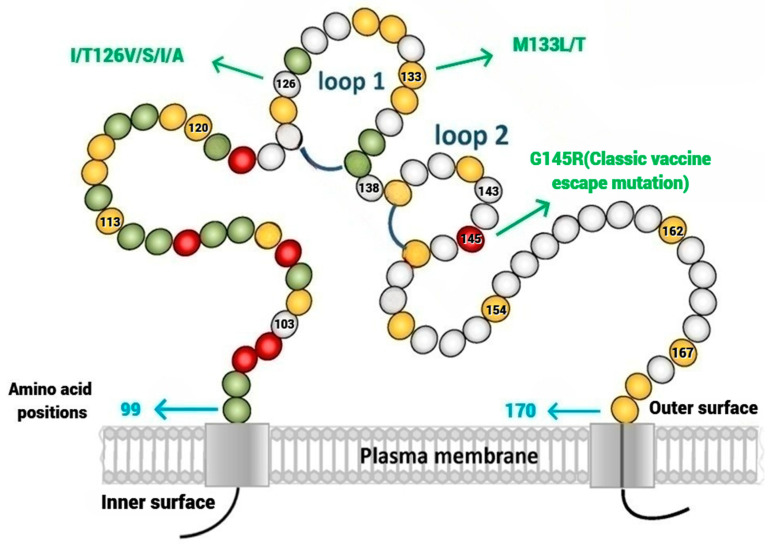
The ‘α’ determinant is a highly conserved region that consists of two loops bounded by disulfide bridges between cys124 and cys137, and cys139 and cys147. Substitutions at specific amino acid positions 126, 133, and 145 are well recognized. The G145R mutation is the classic vaccine-escape mutant.

The HBV genome is a partially double-stranded, relaxed, circular DNA, which consists of four overlapping open-reading frames (ORFs) that code for the Pre-S/S, preCore/Core, Pol, and X genes. The Pre-S/S ORF has the Pre-S1, Pre-S2, and S domains. Based on the starting codon, three hepatitis B surface-antigen (HBsAg) proteins, namely the small (S), middle (M), and large (L), are encoded [3]. HBsAg contains epitopes that are neutralized by antibodies and recognized by T-lymphocytes [4]. The prevalence of Pre-S mutations is highest in HBV genotypes B and C, which are the most common genotypes in Asia [5]. The central core of the small HBsAg (S-HBsAg) consists of the major hydrophilic region (MHR). A cluster of B-cell epitopes within the MHR spanning from amino acid position 124 to position 147 is referred to as the ‘α’ determinant region. It is the antigenic determinant area for the binding of the hepatitis B surface antibody (anti-HBs). The ‘α’ determinant is a highly conserved region that is present in all the HBV genotypes [6,7]. This region consists of two loops bounded by disulfide bridges between cys124 and cys137, and cys139 and cys147 [Figure 1]. Any changes in the amino acid sequence (substitution, deletion, or insertion) in the ‘α’ determinant region can potentially alter the conformational makeup of the HBsAg and can result in the evasion of vaccine-induced immunity, namely the evasion of the anti-HBV immunoglobulin. All these mutations may influence the antigenicity of HBsAg and are thus referred to as ‘immune-escape mutations’. Substitutions at amino acid positions 126, 133, and 145 were most frequently found within the ‘α’ determinant region. The G145R mutation is widely known as the classic HBV vaccine-escape mutation. HBV genotype C was frequently associated with 126 mutations, whereas the 133 and 145 mutations most occurred in genotype B [8].

The isolated presence of HBsAg or hepatitis B antibody (anti-HBs) is associated with ongoing HBV infection or HBV immunity, respectively. The concurrent seropositivity of HBsAg and anti-HBs, however, has been increasingly recognized. The existing literature notes a wide range in terms of the prevalence of co-existent HBsAg and anti-HBs: from 5 to 60%. The recent larger studies, however, reported a rate of less than 10% [9,10,11,12,13,14,15]. It has been proposed that anti-HBs in the setting of concomitant HBsAg (+) and anti-HBs (+) could represent heterotypic antibodies not directed against the common ‘α’ determinant or the circulating HBV serotype. Alternatively, changes in the structure and antigenicity of HBsAg due to HBV S-gene mutations are likely the mechanisms [9,10,11].

The simultaneous presence of an HBsAg (+) and anti-HBs (+) serological pattern was first described over 40 years ago. Its prevalence in regions other than Asia remains unclear, even with the increased rates of HBV vaccination, antiviral treatment, and improved accuracy of diagnostic assays in recent decades. The mutations in the Pre-S/S region, by altering the antigenicity and immunogenicity of HBsAg, can potentially affect polymerase activity, predispose to severe HBV reactivation, and increase risks of hepatocellular carcinoma (HCC) [8,16,17]. Patients with HCC and the co-existence of HBsAg and anti-HBs were noted to have a higher frequency of N-glycosylation mutations in the first loop of S proteins compared to those without [18]. In this study, we systematically evaluated the prevalence, virological, and clinical features of the HBsAg mutations among chronically HBV-infected patients with a concurrent presence of HBsAg and anti-HBs in a cohort of well-characterized patients with chronic hepatitis B (CHB) from the United States.

## 2. Patients and Methods

This is a retrospective study from a single tertiary liver center. A review of the electronic medical record of our HBV serum biorepository from 2011 to 2020 was performed to identify adult CHB patients with concurrent HBsAg (+) and anti-HBs (+). Those with adequate stored sera were selected for analysis. Demographic characteristics, medical history, and laboratory data were collected from the medical records. This study was approved by the institutional review board of Beth Israel Deaconess Medical Center.

HBV DNA was quantitated using Abbott RealTime HBV, and nucleic acid test (NAT) screening assays included Procleix Ultrio and Cobas MPX, with a lower limit of detection of 10 IU/mL. Mutations in Pre-S1, Pre-S2, and S regions from single or serial serum samples (stored at −80 °C) of each patient were determined at Abbott Laboratories as previously described [19]. After DNA extraction, first- and second-round PCR was performed to amplify the Pre-S1-S region using Amplitaq Gold DNA polymerase (Applied Biosystems, Foster City, CA, USA). Amplified products were sequenced. Amino acid substitutions of the gene regions were determined by comparing specimen sequences to the consensus sequence in BioEdit. A *t*-test was used to compare the frequency of mutations in S, Pre-S1, and Pre-S2 gene regions. *p* < 0.05 was considered statistically significant.

## 3. Results

### 3.1. Baseline Demographics and Clinical Features

A total of 2178 CHB patients with stored sera in our biorepository were screened. Among them, 45 (2.1%) were identified to have concurrent reactive HBsAg and anti-HBs. Sequencing data were unavailable for 21 patients due to undetectable HBV DNA (11 patients) or insufficient sera (10 patients). The following analysis was based on the remaining 24 patients, with complete genome sequencing for Pre-S1, Pre-S2, and S regions.

The mean age of these 24 patients was 64.4 (range: 24–76) years, and there were 10 (41.7%) males. The majority, 22 (92%), were Asians who predominantly had genotype B (n = 12) and C (n = 11) [Table 1]. One patient was White with genotype A, and the other patient, of Arab race, had genotype B. Fourteen (58%) patients had a positive family history of hepatitis B and were believed to have either vertical or horizontal transmission of HBV. Eight patients were born and raised in HBV-endemic regions, and two likely acquired HBV via parenteral routes.

At baseline, 20 (83%) of the patients had HBeAg (-) CHB. Two HBeAg (+) patients had HBeAg seroconversion within 1 and 5 years of tenofovir treatment (Table 1). Two Asian females aged 24 and 33 years at baseline remained HBeAg (+) throughout the follow-up period. None of the patients had hepatitis C virus (HCV), hepatitis D virus (HDV), or human immunodeficiency virus (HIV) coinfection. HBV DNA levels were <100 IU/mL in 7, ≥100 to 1999 in 9, and ≥2000 IU/mL in 8 subjects. The median ALT level was 1.23 × Upper Limit Normal (ULN) in this cohort. At baseline, 17 (71%) patients were treatment-naïve, and 7 (29%) were on nuclosis(t)ide analogs. In addition, four patients received antiviral treatment at follow-up, and only 13 patients remained treatment-naïve at the conclusion of the study. Only one patient had documented cirrhosis.

During the median follow-up of 6 years (range: 0–9) from baseline, five (20.8%) patients had HBsAg seroconversion, and two (8%) patients achieved low levels of HBsAg at 0.54 and 4.2 IU/mL, respectively. Of these seven patients, five were treatment-naïve and two received antiviral therapy. None of the 24 patients developed hepatic decompensation or hepatocellular carcinoma. Two of the patients died of liver-unrelated causes. One was due to lymphoma with central nervous system involvement, and the other died due to lung carcinoma. Neither of the two patients had hepatic cirrhosis or decompensation.

### 3.2. Frequencies of Pre-S1, Pre-S2 and S-Gene Mutations

In this cohort, all 24 patients had multiple mutations in these gene regions. They occurred most frequently in the S-gene region compared to the Pre-S1 and Pre-S2 regions. The frequency of mutations in the S gene was significantly higher among the 10 patients older than 50 years, compared to the 14 patients younger than 50 years (mean 8.5 vs. 5.4 mutations per subject, *p* = 0.03). In contrast, the mutation rates were lower and similar in the Pre-S1 (mean 3.2 vs. 3.5, *p* = 0.4) and Pre-S2 (mean 2.2 vs. 2.6, *p* = 0.3) regions for the two age groups. Compared to the 8 patients with HBV DNA ≥2000 IU/mL, the 16 patients with an HBV DNA level < 2000 IU/mL had a higher number of mutations in S (7.8 vs. 5.1 *p* = 0.1), Pre-S1 (4.0 vs. 2.1, *p* = 0.07), and Pre-S2 (2.9 vs. 1.4, *p* = 0.08) regions; however, these differences did not reach statistical significance. The patterns and frequency of gene mutations were similar for those with HBV genotype B [n = 12] and C [n = 11] (S: 7 vs. 7.2, *p* = 0.4; Pre-S1: 2.7 vs. 3.6, *p* = 0.2; Pre-S2: 2.4 vs. 1.7, *p* = 0.2).

### 3.3. Mutations in the ‘α’ Determinant Region (Amino Acid Positions 124–147)

Various mutations have been reported in the ‘α’ determinant region of the HBV, contributing to the diversity of viral strains. Substitutions at specific amino acid positions 126, 133, and 145 are well recognized. The G145R mutation is best characterized as having reduced binding affinity for neutralizing antibodies, thus impacting the effectiveness of the HBV vaccines [20].

In this cohort, 12 (50%) patients had mutations in the ‘α’ determinant region of the S gene (Table 2). The mean age of the patients was 50 (range: 25–75) years, and all but one were Asian. Nine (75%) of the patients were HBeAg (-), and eight (72.7%) had genotype C.

*(A).*  *Mutation at Amino Acid Position 126*

Residue 126 is in the first loop structure of the ‘α’ determinant. In this study, 9 of 12 (75%) patients with mutations in the ‘α’ determinant were found to have mutations in amino acid 126. They were between 24 and 69 years old. Seven patients were female and two were male. All nine patients were Asians; eight had genotype C1/C2 and one had genotype B2. At baseline, only two patients had a DNA level > 2000 IU/mL and one had ALT > 2× ULN. Six out of the nine (66.7%) patients were HBeAg (-), at baseline and an additional patient had HBeAg loss with tenofovir treatment. Three of the nine (33.3%) patients remained treatment-naïve at the last follow-up. One patient achieved HBsAg loss, and this patient was treatment-naïve. One had cirrhosis and none developed hepatic decompensation or HCC.

*(B).*  *Mutation at Amino Acid Position 133*

Three HBeAg (-) subjects aged 42, 72, and 73 years had M133TL variants. Two were Asian and one had Arabic ethnicity. All had genotype B/B2 with HBV DNA < 100 IU/mL at baseline and throughout follow-up, and they were treatment-naïve. The 73-year-old Asian male also had substitutions T131N, F134C, T140S, and T140I. He and the 72-year-old patient of Arabic ethnicity achieved HBsAg loss, and the one remaining patient had an HBsAg level that decreased to 0.54 IU/L at the end of follow-up between 6 and 8 years. None of the patients developed HBV-related complications.

*(C).*  *G145R Vaccine-Escape Mutation*

The classic G145R vaccine-escape mutant was noted in a 29-year-old Asian female with HBeAg-positive genotype C1 infection. This patient received tenofovir and interferon combination therapy for 196 weeks. At baseline, her HBV DNA and ALT were 841 IU/mL and >2× ULN, respectively. Substitution I126T was noted in her sample during early antiviral treatment. She was treated for 4 years initially with tenofovir, a pegylated interferon combination for 1 year, followed by 3 years with tenofovir monotherapy. She achieved HBeAg seroconversion towards the end of therapy. At that time, her DNA was <20 IU/mL with normal ALT. The G145R variant was only detected after HBeAg seroconversion and when DNA was suppressed to <20 IU/mL. Of note, her pretreatment HBsAg was 5792 IU/mL, and it was 4.2 IU/mL after HBeAg seroconversion. She did not receive an HBV vaccine during her therapy and did not develop HBV reactivation after stopping antiviral therapy.

## 4. Discussion

In this study, we found that the prevalence of concurrent HBsAg and anti-HBs was 2.1%; this is consistent with a recent study from the US that reported a prevalence of 1.2% [9]. Our study population and the recent US study population were predominantly Asian. A low prevalence, less than 5%, was also noted in most of the recent studies from Asia with more than 1000 participants [11,12,13,14,15,17]. Eighty-three percent of our cohort with concomitant HBsAg/anti-HBs positivity had HBeAg-negative chronic hepatitis B. There were inconsistent reports regarding the association of HBeAg status with concurrent HBsAg and anti-HBs serology. The majority of the publications reported findings similar to ours [9,10,13,15,17,21]. In contrast, Shiels MT et al., in their 1987 study, noted a high prevalence (32%) of concomitant HBsAg/anti-HBs positivity, and 68% of them were HBeAg-positive. The inclusion of acute hepatitis B and those with intermittently detectable anti-HBs might account for their discrepant results [22].

The Pre-S1 and Pre-S2 regions contain both B- and T-cell epitopes and are highly immunogenic. These mutations were not unique in patients with the coexistence of HBsAg and anti-HBs. Among CHB patients, the prevalence of Pre-S mutations is variable in different geographic areas, with >20% prevalence found in Vietnam, Myanmar, Nepal, and China [5]. Most of these patients had genotypes B and C. In the same study by Huy TT et al., no cases with Pre-S1 and Pre-S2 mutants were seen in Russia, Spain, the United States, and Bolivia. This could be due to the different HBV genotypes in various geographic regions, the number of samples tested, and the stage of infection. The Pre-S1 and Pre-S2 mutations were associated with increased risks of cirrhosis and were believed to play a role in the development of HCC [23,24]. In our cohort, there was only one subject with cirrhosis, and none developed HCC.

In the HBV genome, there is a known overlap between the polymerase gene (P gene) and the surface gene (S gene), so mutations in the Pre-S/S region could lead to amino acid changes in the overlapping polymerase gene, especially in reverse transcriptase (rt). There exists the possibility that the changes in the S gene might confer changes in the P gene that lead to the emergence of splice variants of HBV, which might result in lower or undetectable HBV DNA [25,26]. In our study, when patients were sub-categorized based on DNA levels, those with an HBV DNA level < 2000 IU/mL were associated with increased mutation frequency in Pre-S1, Pre-S2, and S regions compared to patients with DNA > 2000 IU/mL. However, the results did not reach statistical significance.

Similar to the report by Liu Y et al. [27], we found that mutations in the S gene were more frequent than in the Pre-S1 or Pre-S2 regions among those with coexisting HBsAg and anti-HBs. This could be due to the particularly high rate of mutations in the ‘α’ determinant region among those with concurrent HBsAg and anti-HBs compared to those with typical HBV serology. Our study also showed that those older than 50 years had significantly more mutations in the S gene compared to subjects aged 50 or younger, while there was no significant difference in Pre-S region mutations with increasing age. Previous studies alluded to the association of advanced age with a longer duration of HBV infection, leading to increased emergence of immune-escape mutations [28,29]. In this study, 50% of the subjects with coexisting HBsAg and anti-HBs had mutations in the ‘α’ determinant. Consistent with the previous observations [21,27,30,31], point mutations at amino acids 126 and 133, in addition to 145 vaccine-escape mutations, were most frequently identified. These mutations are often present as occult hepatitis B since some commercial assays may not be able to detect the HBsAg associated with these mutations [30]. Severe HBV reactivation in patients who were HBsAg-negative has been reported as a consequence of these mutations, especially in the setting of immunosuppression.

Residue 126 is in the first loop structure of the ‘α’ determinant, and mutation here may result in the evolution of HBV infection, leading to a change in HBsAg antigenicity and virulence. We observed the highest frequency of mutations in amino acid 126 compared to other sites in the ‘α’ determinant region. The majority (87%) of the patients with mutations at site 126 were genotype C. This association with genotype C is consistent with prior studies [21,27,31]. Fu et al. also noted that the amino acid variability in the first loop is significantly higher than the second loop of the ‘α’ determinant region [21]. There were reports stating that mutation I126S occurred frequently in HBV isolates, which might represent a polymorphism among the HBV genotype C isolates [10,32,33]. Yan B et al. [34] reported the temporal trend in the prevalence and evolution of *S*-gene mutations in the post-immunization era in China from 2005 to 2013. The most frequent mutation positions were amino acids 126 and 145. While the prevalence of ‘α’ mutations showed no significant change during the consecutive 9 years of the massive vaccine period, the frequency of residue 126 mutations decreased, and mutations at residue 145 were more frequently observed, especially in the years between 2011 and 2013. The authors speculated that the substitutions in amino acid position 145 were under greater anti-HB immune pressure, associated with increasing massive vaccination compared to the other epitope sites of the ‘α’ determinant region.

The classic vaccine-escape mutant, G145R, was only observed in one of our patients. She was a young Asian female with genotype C1. This relatively low frequency is consistent with some previous studies on concomitant HBsAg and anti-HBs [12,17,27]. Interestingly, in her clinical course, the I126T mutation was noted in her early treatment sample, and the G145R mutation was only detected after HBeAg clearance with suppressed HBV DNA. It is possible that the viral heterogeneity increased over time and selective pressure favored the replication of G145R even though it was most frequently associated with genotype B [35].

Mutation at amino acid position 133 with the substitution of methionine for isoleucine could result in occult hepatitis B [36,37]. Similarly, the substitution of methionine for threonine was reported to cause acute de novo post-transplant hepatitis with severe liver dysfunction in a previously healthy patient with nonreactive HBsAg and reactive anti-HBs with a high titer of 256 IU/l. This patient also had asparagine to threonine at amino acid position 131 [38]. In our study, three genotype B patients had a mutation at amino acid 133, where methionine was substituted with leucine or threonine. All three had HBeAg-negative chronic infection with low HBsAg and HBV DNA levels. After 6 to 8 years of follow-up, none developed HBV reactivation or disease progression. In fact, two achieved HBsAg clearance. It is still important to note that these mutations have the potential to cause severe HBV reactivation, especially under immunosuppression.

In this retrospective study, our observations confirmed the findings of many recent publications from predominantly Asian populations with HBV genotypes B and C. Our study has its limitations. First, the sample size for Pre-S/S-gene sequencing was small, and we did not include patients with HBsAg-positive and anti-HBs-negative chronic hepatitis B as controls. Secondly, we included patients who received antiviral therapy after the baseline samples. The severity of the liver disease, change in viral replication, or HBV reactivation in follow-up could not be assessed for the treated patients.

In conclusion, we found a relatively low prevalence of concomitant HBsAg and anti-HBs among this predominantly HBeAg-negative Asian population in a tertiary liver center in the United States. The S-gene mutations were more frequent compared to those in the Pre-S1 and Pre-S2 regions, and they were more frequent with advancing age. Approximately 50% of the S-gene mutations were identified in the ‘α’ determinant region. Despite the universal HBV vaccination practice and increased nucleos(t)ide treatment, the classical HBV vaccine-escape mutant was rare in our cohort. It is important to recognize that patients with S-gene mutations in the ‘α’ determinant region could develop severe HBV reactivation under immunosuppression.

## Figures and Tables

**Table 1:** Baseline characteristics of the 24 patients.

Patients with Available Sequencing Data	24/45 (53%)
Age, years	64.4 (24–76)
Gender, n (%)	
Male	10 (41.7%)
Female	14 (58.3%)
Race, n (%)	
Asian	22 (92%)
Arab	1 (4%)
White	1 (4%)
HBeAg, n (%)	
Positive	4 (16.7%)
Negative	20 (83.3%)
Genotype, n (%)	
A	1 (4%)
B	12 (50%)
C	11 (46%)
HBV Treatment, n (%)	
Yes	7 (29%)
No	17 (71%)
HBV DNA, n (%)	
<100 IU/mL	7 (29.2%)
>100–2000 IU/mL	9 (37.5%)
>2000 IU/mL	8 (33.3%)
Cirrhosis, n (%)	1 (4%)

**Table 2:** Characteristics of patients with mutations in ‘α’ determinant region.

Case no.	Year Since Baseline	Age	Gender	Race	HBV DNA (IU/mL)	ALT (IU/mL)	HBeAg	HBsAgNx (S/CO)	Treatment	‘α’ Determinant Region Mutations
6	0.0	25	F	Asian	841	68	pos	5792	tenofovir and interferon	
	0.7	26			2130	17		3364	tenofovir and interferon	I126T
	4.1	29			<20	11	neg	515	off-meds	G145R
	7	32			16	7	neg	4.2 *	off-meds	not available
4	0.0	42	F	Asian	68	43	neg	287	naïve	M133L
	4.2	46			20	27		150	naïve	M133L
	8.0	50			<LLOD	13		0.54 *	naïve	not available
22	0	72	M	Arab	78	15	neg	4045	naïve	M133L
	6.6	78			<LLOD	31		(-)		not available
24	0.0	73	M	Asian	126	15	neg	42	naïve	F134C, T140S
	1.7	74			22	20		12	naïve	T131N, M133T, T140I
	6.0	81			<LLOD	18		(-)		not available
2	0.0	35	F	Asian	1780	42	pos	5870	off tenofovir therapy	I126V
	7.0	42			<LLOD	16		1813		not available
3	0.0	63	F	Asian	3093	13	neg	4538	naïve	I126M
	8.0	71			170	12		(+)	naïve	not available
7	0.0	40	M	Asian	1513	66	neg	5944	tenofovir	I126S
	9.0	49			<LLOD	15		796	vemlidy	not available
8	1.9	70	F	Asian	<20	21	neg	6181	on adefovir	T126A
	3.0	71			145	27		(+)		not available
12	0.3	34	F	Asian	<40	52	neg	3573	naïve	I126S
14	0.0	24	F	Asian	>110 million	21	pos	4851	naive	I126S
	4.7	29			UA	38		5776	naive	I126S
	7.3	32			UA	75		4550	naive	I126S
	8.0	33			<LLOD	11		13788	vemlidy	not available
16	0.0	69	F	Asian	26	44	neg	4904	naive	I126T
	5.0	74			<LLOD	20		<0.05	naive	not available
18	0.0	53	M	Asian	<20	73	neg	10	naive	I126S
	2.8	56			UA	25		5191	on vemlidy	I126T
	6.0	59			<LLOD	26		(+)	tenofovir	not available

* qHBsAg was done in Quest Diagnostics and was reported as IU/mL.

## Data Availability

Data are contained within the article and Supplementary Materials.

## Supplementary Materials

The following supporting information can be downloaded at: https://www.mdpi.com/article/10.3390/v16050713/s1, Table S1: Clinical and Virological Characteristics of 24 patients with available gene sequencing; Table S2: Pre-S1, Pre-S2, S-gene mutations for the 24 patients and the consensus sequences.
