# Supplementary material for: Immune-Escape Mutations Are Prevalent among Patients with a Coexistence of HBsAg and Anti-HBs in a Tertiary Liver Center in the United States
Source: Viruses. 2024 Apr 30;16(5):713. doi: 10.3390/v16050713 (PMC11125813; doi:10.3390/v16050713)
Supplement: Supplementary file 1 [file viruses-16-00713-s001.zip › viruses-2952228-supplementary.pdf]

Supplemental Table S1:

Clinical and Virological Characteristics of 24 patients with available gene sequencing

Case no.	Years since baseline	Age, years	Phenotype	HBV DNA (IU/mL)	ALT (IU/L)	HBsAgNx (S/CO)
1	0.0	60	HBeAg (-) (indeterminant)	326	41	359
	2.0	62	HBeAg (-) chronic infection	275	27	310
	4.2	64		1180	21	324
	7.0	67		200	23	20
	9.0	69		32	19	(+)
2	0.0	35	HBeAg (+) on antiviral therapy	1780	42	5870
	2.1	37		20	14	6080
	4.7	40		10	14	5633
	7.0	42		<LLOD	16	1813*
3	0.0	63	HBeAg (-) (indeterminant)	3093	13	4538
	5.2	68		3981	14	5801
	8.0	71	HBeAg (-) chronic infection	170	12	(+)
4	0.0	42	HBeAg (-) (indeterminant)	68	43	287
	4.2	46	HBeAg (-) chronic infection	20	27	150
	6.2	48		50	16	274
	8.0	50		<LLOD	13	0.54*
5	0.0	33	HBeAg (-) (indeterminant)	2985	13	1153
	1.0	34	HBeAg (-) chronic infection	<LLOD	7	not available
6	0.0	25	HBeAg (+) on antiviral therapy	841	68	5792
	0.1	25		150	46	5239
	0.7	26		2130	17	3364
	0.8	26	HBeAg (-) chronic infection	<20	16	3404

	1.8	27		<LLOD	15	2575
	3.9	29		<20	13	296
	4.1	29		<20	11	515
	4.2	30		<20	10	286
	7.0	32		16	7	4.2*
7	0.0	40	HBeAg (-) on antiviral therapy	1513	66	5944
	3.7	44		<20	57	6135
	5.7	46		<20	46	5549
	9.0	49		<LLOD	15	796*
8	0.0	68	HBeAg (-) on antiviral therapy	65	26	6232
	1.9	70		<20	21	6181
	3.0	71		145	27	(+)
9	0.0	34	HBeAg (-) on antiviral therapy	<20	47	5718
	2.5	36		877	19	5315
	3.4	37		<20	30	5743
	4.0	38		<LLOD	23	(-)
10	0.0	29	HBeAg (-) on antiviral therapy	2351	13	4349
	1.8	31		22900	25	2877
	5.2	34		500	16	627
	8.0	37		3.2	16	(-)
11	0.0	41	HBeAg (-) chronic infection	1470	33	6033
	0.0	52	No available	not available	12	not available
12	0.0	34	HBeAg (-) (indeterminant)	<40	46	4304
	0.3	34	HBeAg (-) (indeterminant)	<40	52	3573
	0.6	34	HBeAg (-) chronic infection	60	28	4129
	0.8	35	not available	not available	not available	not available

13	0.0	69	HBeAg (-) chronic infection	704	21	6082
	1.1	70		494	21	6332
	2.0	71		<LLOD	32	not available
14	0.0	24	HBeAg (+) chronic infection	>110 million	21	4851
	4.7	29	HBeAg (+) on anti-viral therapy	UA	38	5776
	7.3	32		UA	75	4550
	8.0	33		<LLOD	11	13788*
15	0.0	33	HBeAg (+) on anti-viral therapy	178851	32	4178
	0.0	36		>170	25	(+)
16	0.0	69	HBeAg (-) (indeterminate)	26	44	4904
	2.0	71	HBeAg (-) chronic infection	<LLOD	34	3.45
	5.0	74		<LLOD	20	(-)
17	0.0	74	HBeAg (-) chronic infection	144	20	1741
	3.6	77		26	16	288
	4.0	80		<10	11	(+)
18	0.0	53	HBeAg (-) (indeterminate)	<20	73	10
	2.8	56	HBeAg (-) on antiviral therapy	UA	25	5191
		59		<LLOD	26	(+)
19	0.0	34	HBeAg (-) (indeterminate)	12,590	35	4822
		-	not available	not available	not available	not available
20	0.0	48	HBeAg (-) chronic hepatitis	125, 890	39	5355
		50		<LLOD	25	not available

21	0.0	31	HBeAg (-) chronic hepatitis	79,432,823	41	2835
		-		not available	not available	not available
22	0.0	72	HBeAg (-) chronic infection	78	15	4045
	6.0	78		<LLOD	31	(-)
23	0.0	76	HBeAg (-) chronic infection	320	17	109
	0.7	76		66	15	39
	0.8	77		<20	15	3.2
	3.0	79		UD	13	1.95
	3.4	79		UD	24	5.91
	6.0	80		UD	14	(-)
24	0.0	73	HBeAg (-) chronic infection	126	15	42
	1.7	74		22	20	12
	6.0	81		<LLOD	18	(-)

* qHBsAg done by Quest Diagnostics Laboratories. It reported as IU/mL

Supplemental Table S2 **Pre-S1, Pre-S2, S-gene mutations for the 24 patients and the consensus sequences.**

Patient number on supplemental Table 1	preS1-S PCR	seq #	HBV Genotype	PreS1 Mutations	PreS2 Mutations	S Mutations, vs genotype consensus
1	pos	2	B4	T68I, L84I, A91T	S5T, V39A, I45T, L46F	V14AV, C64CY, L95W, K122R, Y161F, F200Y, Y206C, I208IT
	pos	3a	B4	T68I, L84I, A91T	S5T, V39A, I45T, L46F	F80S, K122R, F158S, Y161F, F200Y, Y206C, I208T, F220C
	pos	3b	B4	K35Q, E39G, T68I, L84I, A91T	S5T, V39A, I45T, L46F	V14A,, F80FS, K122R, F158S, Y161F, F200Y, I208T
	pos	4	B4	T68IT, L84I, A91T	S5T, H9P, V39A, I45T, L46F	V14AV, C64CY, L95W, K122R, Y161F, A166G, F200Y, Y206CY, I208IT
	pos	5a	B4	T68I, L84I, A91T, N98HN	S5T, V39A, I45T, L46F	V14AV, S34L, C64CY, L95W, K122R, Y161F, F200Y, Y206CY, I208IT
	pos	5b	B4	T68IT, L84I, A91T	S5T, V39A, I45T, L46F	V14AV, L95W, K122R, Y161F, F200Y, S204N
2	pos	8a	C2	S38T, A90S	None	I28M, I126V
	pos	8b	C2	A90S	None	I126V
3	pos	11a	C1	D27G, H51Q, E54A, A60V, A62S, G73N	T6S, N55S	G18V, L26H, P29L, S31N, T57I, R79H, L98V, S204N, S210T
	pos	11b	C1	D27G, H51Q, E54A, A60V, A62S, G73N	T6S	N3S, I92T, I126IM, G202A, Y206H, S210N, P211H, L216F
	pos	12	C1	D27G, H51Q, E54A, A60V, A62S, G73N	T6S	N3S, V14GV, G18V, I92T, G202A, Y206H, S210N, P211H, L216F
4	pos	16a	B2	P19T, E39A, L84I	M1I, Q2R	N40S, G44E, **M133L,** Y161F, F200Y, Y206C
	pos	16b	B2	P19T, E39A, L84I	M1IM, Q2RQ, A19DA	N40NS, G44E, **M133L**, Y161F, F200Y, Y206C
	pos	17	B2	P8T, T14I, P19T, E39A, L84I	M1I, Q2R, A19D	N40S, G44E, **M133L**, Y161F, F200Y, Y206C, F220C
5	pos	19	B2	P65T, L84I	None	F19Y, I25V, G44D, L49LP, C85CF, Y161FY, F200Y, S204R, M213I
6	pos	27	C1	Q10QK, P32L, G35R, N39ND, H51P, N56H, A60V, A62S, G73N, V88VI	T6S, S28N, I42T	N3S, S53L, I126T, V177A, S210N
7	pos	33a	C1	Q10K, P26G, A33T, G35R, F45R, E54A, A60V, A62S, G73N	T6S, S29F	N3S, R24K, S53L, I92T, V96G, Q101K, T115N, **I126S**, S210N
	pos	33b	C1	Q10K, P26G, A33T, G35R, F45R, E54A, A60V, A62S, G73N, P93A	T6S, S29F	N3S, R24K, S53L, I92T, L94LS, Q101K, T115N, **I126S**, S210N
	neg	34	neg			
	neg	35	neg			
8	pos	37	B2	F67V	W3G	A5S, L21S, G44E, T126A, M213I
9	pos	39	B4	K10Q, D54N, L84I	V39A, I45T, L46F	K122R, F200Y, M213I
10	pos	41	B2	T68I, L84I	Q36P	L21LS, F200Y
	pos	42	B2	T68I, L84I	Q36P	F200Y
	pos	43	B2	T68I, L84I	Q36P	V106A, F200Y, M213MT
11	pos	46	A1	A54Q, L67F, I74V, T86A, S89P, T90A, I91V	M1IM, F8LF, L12R, P15P, R16K, Y21C, V32L, A35V, A47S, V53A	S53LS, **Y100C,** S207N, V209L
12	pos	50	C2	L74I	R48K	L95W, **I126S**
	pos	51	C2	None	T6P, T7K, R16K, L20Q, Y21N, F22L	M1T, L13H, F20S, W74L, L77R, V96A, L98V, T113S, S114T, T115N, G119L, **P120S,** R160K, I195T, Y206N, P214L, C221Y
13	pos	52	C2	G35R	None	N3S, N40S, G44E, P46L, T47K, S64C, I68T, V184A, Y200L, L213T
	pos	53	C2	G35R	None	N3S, N40S, G44E, P46L, T47K, S64C, I68T, V184A, Y200L, F212CF, L213T
14	pos	54	C2	L74I	None	L95LW, **I126IS**
	pos	55	C2	L74I	None	L95LW, **I126S**
	pos	56	C2	L74I	DEL7-21, F22I	P62LP, L95LW, **I126IS**
15	pos	57	C2	G73S	None	V184A
16	pos	58	C2	A90V	None	T47A, I126T, V194A, Y200F
	pos	59	C2	A90V, L112M	None	L84F, V194A, Y200F
17	pos	60	B2	None	None	N40S, V47E, M197MT, Y206C, F220C
	pos	61	B2	None	None	N40S, V47E, S58C, Y206C,F220C
18	pos	62a	C2	L74I	R16G	L95W, **I126S**
	pos	62b	C2	L74I	DEL7-21, F22I	P62L
	pos	63	C1	H51Q, E54A, N56H, A60V, A62S, G73N	M1I, T6S, T7K, V17P	R24K, S53L, P56LP, T118A, I126T, G202EG, I208IT, S210N
19	pos	64	C2	None	None	G44E, A45T, F80S, F85C, I92T, S204R, L209W, P214H
20	neg	69	B4	H48Y, L84I	V39A, I45T, L46F	S55FS, C76Y, K122R, M198I, F200Y, M213I
21	pos	70	B4	N51T,T68I, L84I	V39A, I45T, L46F	K122R, F200Y
22	pos		B2	P65T, L84I, S101T	None	**M133L**, F200Y, L216F
23	pos		B2	L85F	K48KT	S31SN, N40S, G44GE, V47E, L49LP, I68IT, A159V, Y206C, I218IL, F220FL
	pos		B2	L85F, S109T	S44L,T49TI, P52L, P54T	N40S, V47E, L94LS, Y100FS, Q101QK, A159V, I208IT, F220L
24	pos		B	E44D, A60S, L84I,S101L	S33N, A35V,V39A, A41S, V53A	S113P, T116N, K122R, F134C, T140S, A159V, L175S, S204N, N207R
	pos		B	L84I, D49DN, S101L, A60S, L84I,S101L	Q10K, S33N, A35V,V39VA, A41S, V53A	F41FS, G44A, V47E, Q51QL, S59SN, I68IT, K122R, **T131N, M133T,** T140I, F200L,

```
                           10        20        30        40        50        60
                   ....|....|....|....|....|....|....|....|....|....|....|....|
Consensus A preS1  MGGWSSKPRKGMGTNLSVPNPLGFFPDHQLDPAFGANSNNPDWDFNPIKDHWPAANQVGV
A1-46a-1201        .....................................................Q......
A1-46b-1201        .....................................................Q......

                           70        80        90        100       110
                   ....|....|....|....|....|....|....|....|....|....|....|....
Consensus A preS1  GAFGPGLTPPHGGILGWSPQAQGILTTVSTIPPPASTNRQSGRQPTPISPPLRDSHPQA
A1-46a-1201        ......F......V...........A..PAV............................
A1-46b-1201        ......F......V...........A..PAV............................

                           10        20        30        40        50        60
                   ....|....|....|....|....|....|....|....|....|....|....|....|
Consensus B preS1  MGGWSSKPRKGMGTNLSVPNPLGFFPDHQLDPAFKANSENPDWDLNPHKDNWPDANKVGV
B4-2a-1201         ............................................................
B4-2b-1201         ............................................................
B4-3a-1201         ............................................................
B4-3b-1201         ..................................Q...G.....................
B4-4a-1201         ............................................................
B4-4b-1201         ............................................................
B4-5a-1201         ............................................................
B4-5b-1201         ............................................................
B2-16a-1201        ..................T...................A.....................
B2-16b-2101        ..................T...................A.....................
B2-17b-1201        .......T.....I....T...................A.....................
B2-18b-1201        .................LT.S.................A.....................
B2-19b-1201        ............................................................
B2-37b-1201        ............................................................
B4-39a-1201        .........Q...........................................N......
B4-39b-1201        .........Q...........................................N......
B2-41a-1201        ............................................................
B2-41b-1201        ............................................................
B2-42a-1201        ............................................................
B2-42b-1201        ............................................................
B2-43a-1201        ............................................................
B2-43b-1201        ............................................................
B2-60a-1201        ............................................................
B2-60b-1201        ............................................................
B2-61a-1201        ............................................................
B2-61b-1201        ............................................................
B4-70a-1201        ..................................................T.........
B4-70b-1201        ..................................................T.........

                           70        80        90        100       110
                   ....|....|....|....|....|....|....|....|....|....|....|....
Consensus B preS1  GAFGPGFTPPHGGLLGWSPQAQGLLTTVPAAPPPASTNRQSGRQPTPLSPPLRDTHPQA
B4-2a-1201         .......I...............I......T............................
B4-2b-1201         .......I...............I......T............................
B4-3a-1201         .......I...............I......T............................
B4-3b-1201         .......I...............I......T............................
B4-4a-1201         .......I...............I......T............................
B4-4b-1201         .......I...............I......T............................
B4-5a-1201         .......I...............I......T......H.....................
B4-5b-1201         .......I...............I......T............................
B2-16a-1201        .......................I...................................
B2-16b-2101        .......................I...................................
B2-17b-1201        .......................I...................................
B2-18b-1201        .......................I...................................
B2-19b-1201        ....T..................I...................................
B2-37b-1201        ......V....................................................
B4-39a-1201        .......................I...................................
```

```
B4-39b-1201       .......................I...................................
B2-41a-1201       .......I...............I...................................
B2-41b-1201       .......I...............I...................................
B2-42a-1201       .......I...............I...................................
B2-42b-1201       .......I...............I...................................
B2-43a-1201       .......I...............I...................................
B2-43b-1201       .......I...............I...................................
B2-60a-1201       ...........................................................
B2-60b-1201       ...........................................................
B2-61a-1201       ...........................................................
B2-61b-1201       ...........................................................
B4-70a-1201       .......I...............I...................................
B4-70b-1201       .......I...............I...................................
```

```
                          10        20        30        40        50        60
                  ....|....|....|....|....|....|....|....|....|....|....|....|
Consensus C preS1 MGGWSSKPRQGMGTNLSVPNPLGFFPDHQLDPAFGANSNNPDWDFNPNKDHWPEANQVGA
C2-8a-1201        .....................................T......................
C2-8b-1201        ............................................................
C1-11a-1201       ..........................G.......................Q..A.....V
C1-11b-1201       ..........................G.......................Q..A.....V
C1-12b-1201       ..........................G.......................Q..A.....V
C1-27b-1201       .........K.....................L..R...D...........P....H...V
C1-31a-1201       ...............................L..R...............Q..A.H...V
C1-31b-1201       ...............................L..R...............Q..A.H...V
C1-33a-1201       .........K...............G......T.R.........R........A.....V
C1-33b-1201       .........K...............G......T.R.........R........A.....V
C2-50a-1201       ............................................................
C2-50b-1201       ............................................................
C2-51a-1201       ............................................................
C2-51b-1201       ............................................................
C2-52a-1201       ..................................R.........................
C2-52b-1201       ..................................R.........................
C2-53a-1201       ..................................R.........................
C2-53b-1201       ..................................R.........................
C2-54a-1201       ............................................................
C2-54b-1201       ............................................................
C2-55a-1201       ............................................................
C2-55b-1201       ............................................................
C2-56a-1201       ............................................................
C2-56b-1156       ............................................................
C2-57a-1201       ............................................................
C2-57b-1201       ............................................................
C2-58b-1201       ............................................................
C2-59b-1201       ............................................................
C2-62a-1201       ............................................................
C2-62b-1156       ............................................................
C1-63a-1201       ..................................................Q..A.H...V
C2-64a-1201       ............................................................
C2-64b-1201       ............................................................
```

```
                            70        80        90       100       110
                  ....|....|....|....|....|....|....|....|....|....|....|....
Consensus C preS1 GAFGPGFTPPHGGLLGWSPQAQGILTTVPAAPPPASTNRQSGRQPTPISPPLRDSHPQA
C2-8a-1201        .............................S.............................
C2-8b-1201        .............................S.............................
C1-11a-1201       .S..........N..............................................
C1-11b-1201       .S..........N..............................................
C1-12b-1201       .S..........N..............................................
```

```
C1-27b-1201          .S..........N..............I...............................
C1-31a-1201          .S..........N..............................................
C1-31b-1201          .S..........N..............................................
C1-33a-1201          .S..........N..............................................
C1-33b-1201          .S..........N...................A..........................
C2-50a-1201          .............I.............................................
C2-50b-1201          .............I.............................................
C2-51a-1201          ...........................................................
C2-51b-1201          ...........................................................
C2-52a-1201          ...........................................................
C2-52b-1201          ...........................................................
C2-53a-1201          ...........................................................
C2-53b-1201          ...........................................................
C2-54a-1201          .............I.............................................
C2-54b-1201          .............I.............................................
C2-55a-1201          .............I.............................................
C2-55b-1201          .............I.............................................
C2-56a-1201          .............I.............................................
C2-56b-1156          .............I.............................................
C2-57a-1201          ............S..............................................
C2-57b-1201          ............S..............................................
C2-58b-1201          .............................V.............................
C2-59b-1201          .............................V.....................M.......
C2-62a-1201          .............I.............................................
C2-62b-1156          .............I.............................................
C1-63a-1201          .S..........N..............................................
C2-64a-1201          ...........................................................
C2-64b-1201          ...........................................................
```

Highlighted: mixture WT/mutant at 50%

```
                            10        20        30        40        50
                   ....|....|....|....|....|....|....|....|....|....|....|
Consensus A preS2  MQWNSTAFHQALQDPRVRGLYFPAGGSSSGTVNPAPNIASHISSISARTGDPVTN
A1-46a-1201        I......L...R..TK....C..........L..V...........S.....A..
A1-46b-1201        I......L...R..TK....C..........L..V...........S.....A..

                            10        20        30        40        50
                   ....|....|....|....|....|....|....|....|....|....|....|
Consensus B preS2  MQWNSTTFHQTLQDPRVRALYFPAGGSSSGTVSPAQNTVSAISSILSKTGDPVPN
B4-2a-1201         ....T.................................A.....TF.........
B4-2b-1201         ....T.................................A.....TF.........
B4-3a-1201         ....T.................................A.....TF.........
B4-3b-1201         ....T.................................A.....TF.........
B4-4a-1201         ....T.................................A.....TF.........
B4-4b-1201         ....T...P.............................A.....TF.........
B4-5a-1201         ....T.................................A.....TF.........
B4-5b-1201         ....T.................................A.....TF.........
B2-16a-1201        IR.....................................................
B2-16b-2101        IR................D....................................
B2-17b-1201        IR................D....................................
B2-18b-1201        IR.....................................................
B2-19b-1201        .......................................................
B2-37b-1201        ..G....................................................
B4-39a-1201        ......................................A.....TF.........
B4-39b-1201        ......................................A.....TF.........
B2-41a-1201        ...................................P...................
B2-41b-1201        ...................................P...................
B2-42a-1201        ...................................P...................
B2-42b-1201        ...................................P...................
B2-43a-1201        ...................................P...................
B2-43b-1201        ...................................P...................
B2-60a-1201        .......................................................
B2-60b-1201        .......................................................
B2-61a-1201        .......................................................
B2-61b-1201        .......................................................
B4-70a-1201        ......................................A.....TF.........
B4-70b-1201        ......................................A.....TF.........
```

```
                            10        20        30        40        50
                    ....|....|....|....|....|....|....|....|....|....|....|
Consensus C preS2   MQWNSTTFHQALLDPRVRGLYFPAGGSSSGTVNPVPTTASPISSIFSRTGDPAPN
C2-8a-1201          .......................................................
C2-8b-1201          .......................................................
C1-11a-1201         .....S................................................S
C1-11b-1201         .....S.................................................
C1-12b-1201         .....S.................................................
C1-27b-1201         .....S.....................N.............T.............
C1-31a-1201         .....S.................................................
C1-31b-1201         .....S.................................................
C1-33a-1201         .....S......................F..........................
C1-33b-1201         .....S......................F..........................
C2-50a-1201         ...............................................K.......
C2-50b-1201         .......................................................
C2-51a-1201         .....PK........K...QNL.................................
C2-51b-1201         .....PK........K...QNL.................................
C2-52a-1201         .......................................................
C2-52b-1201         .......................................................
C2-53a-1201         .......................................................
C2-53b-1201         .......................................................
C2-54a-1201         .......................................................
C2-54b-1201         .......................................................
C2-55a-1201         .......................................................
C2-55b-1201         .......................................................
C2-56a-1201         ......~~~~~~~~~~~~~~~I.................................
C2-56b-1156         ......~~~~~~~~~~~~~~~I.................................
C2-57a-1201         .......................................................
C2-57b-1201         .......................................................
C2-58b-1201         .......................................................
C2-59b-1201         .......................................................
C2-62a-1201         ...............G.......................................
C2-62b-1156         ......~~~~~~~~~~~~~~~I.................................
C1-63a-1201         I....SK.........P.....................................K
C2-64a-1201         .......................................................
C2-64b-1201         .......................................................
```

Highlighted: mixture WT/mutant at 50%

```
                           10        20        30        40        50        60
                   ....|....|....|....|....|....|....|....|....|....|....|....|
Consensus genA_S   MENITSGFLGPLLVLQAGFFLLTRILTIPQSLDSWWTSLNFLGGSPVCLGQNSQSPTSNH
A1-46a-1201        ....................................................L.......
A1-46b-1201        ....................................................L.......

                           70        80        90       100       110       120
                   ....|....|....|....|....|....|....|....|....|....|....|....|
Consensus genA_S   SPTSCPPICPGYRWMCLRRFIIFLFILLLCLIFLLVLLDYQGMLPVCPLIPGSTTTSTGP
A1-46a-1201        .......................................C....................
A1-46b-1201        .......................................C....................

                          130       140       150       160       170       180
                   ....|....|....|....|....|....|....|....|....|....|....|....|
Consensus genA_S   CKTCTTPAQGNSMFPSCCCTKPTDGNCTCIPIPSSWAFAKYLWEWASVRFSWLSLLVPFV
A1-46a-1201        ............................................................
A1-46b-1201        ............................................................

                          190       200       210       220
                   ....|....|....|....|....|....|....|....|..
Consensus genA_S   QWFVGLSPTVWLSAIWMMWYWGPSLYSIVSPFIPLLPIFFCLWVYI*
A1-46a-1201        ............L............FNTL.............
A1-46b-1201        ..........................N.L.............

                           10        20        30        40        50        60
                   ....|....|....|....|....|....|....|....|....|....|....|....|
Consensus B S      MENIASGLLGPLLVLQAGFFLLTKILTIPQSLDSWWTSLNFLGGTPVCLGQNSQSQISSH
B4-2a-1201         .............A..............................................
B4-2b-1201         .............A..............................................
B4-3a-1201         ............................................................
B4-3b-1201         .............A..............................................
B4-4a-1201         .............A..............................................
B4-4b-1201         .............A..............................................
B4-5a-1201         .............A...................L..........................
B4-5b-1201         .............A..............................................
B2-16a-1201        .......................................S...E................
B2-16b-2101        .......................................S...E................
B2-17b-1201        .......................................S...E................
B2-18b-1201        .......................................S...E................
B2-19b-1201        ..................Y.....V..................D....P...........
B2-37b-1201        ....S...............S......................E................
B4-39a-1201        ....................S..................S...E................
B4-39b-1201        ............................................................
B2-41a-1201        ....................S.......................................
B2-41b-1201        ....................S.......................................
B2-42a-1201        ............................................................
B2-42b-1201        ............................................................
B2-43a-1201        ............................................................
B2-43b-1201        ...........................................E................
B2-60a-1201        .......................................S......E.............
B2-60b-1201        .......................................S......E.............
B2-61a-1201        .......................................S......E..........C..
B2-61b-1201        .......................................S.....TE..........C..
B4-70a-1201        ............................................................
B4-70b-1201        ............................................................
```

```
                         70        80        90       100       110       120
                 ....|....|....|....|....|....|....|....|....|....|....|....|
Consensus B S    SPTCCPPICPGYRWMCLRRFIIFLCILLLCLIFLLVLLDYQGMLPVCPLIPGSSTTSTGP
B4-2a-1201       ..................................W.........................
B4-2b-1201       ...Y..............................W.........................
B4-3a-1201       ...................S........................................
B4-3b-1201       ...................S........................................
B4-4a-1201       ...Y..............................W.........................
B4-4b-1201       ...Y..............................W.........................
B4-5a-1201       ...Y..............................W.........................
B4-5b-1201       ..................................W.........................
B2-16a-1201      ............................................................
B2-16b-2101      ............................................................
B2-17b-1201      ............................................................
B2-18b-1201      ............................................................
B2-19b-1201      ........................F...................................
B2-37b-1201      ............................................................
B4-39a-1201      L...........................................................
B4-39b-1201      ............................................................
B2-41a-1201      ............................................................
B2-41b-1201      ............................................................
B2-42a-1201      ............................................................
B2-42b-1201      ............................................................
B2-43a-1201      .............................................A..............
B2-43b-1201      .............................................A..............
B2-60a-1201      ............................................................
B2-60b-1201      ............................................................
B2-61a-1201      ............................................................
B2-61b-1201      ............................................................
B4-70a-1201      ............................................................
B4-70b-1201      ............................................................
```

```
                        130       140       150       160       170       180
                 ....|....|....|....|....|....|....|....|....|....|....|....|
Consensus B S    CKTCTTPAQGTSMFPSCCCTKPTDGNCTCIPIPSSWAFAKYLWEWASVRFSWLSLLVPFV
B4-2a-1201       .R......................................F...................
B4-2b-1201       .R......................................F...................
B4-3a-1201       .R.....................................S..F...................
B4-3b-1201       .R.....................................S..F...................
B4-4a-1201       .R......................................F...................
B4-4b-1201       .R......................................F....G..............
B4-5a-1201       .R......................................F...................
B4-5b-1201       .R......................................F...................
B2-16a-1201      ...........L............................F...................
B2-16b-2101      ...........L............................F...................
B2-17b-1201      ...........L............................F...................
B2-18b-1201      ........................................F...................
B2-19b-1201      ........................................F...................
B2-37b-1201      .....A......................................................
B4-39a-1201      .R..........................................................
B4-39b-1201      .R..........................................................
B2-41a-1201      ............................................................
B2-41b-1201      ............................................................
B2-42a-1201      ............................................................
B2-42b-1201      ............................................................
B2-43a-1201      ............................................................
B2-43b-1201      ............................................................
B2-60a-1201      ............................................................
B2-60b-1201      ............................................................
B2-61a-1201      ............................................................
B2-61b-1201      ............................................................
B4-70a-1201      .R..........................................................
```

```
B4-70b-1201     .R..........................................................

                        190       200       210       220
                ....|....|....|....|....|....|....|....|....|..
Consensus B S   QWFVGLSPTVWLSVIWMMWFWGPSLYNILSPFMPLLPIFFCLWVYI*
B4-2a-1201      ...................Y.....C.T..............
B4-2b-1201      ...................Y.....C.T..............
B4-3a-1201      ...................Y.....C.T...........C..
B4-3b-1201      ...................Y.......T..............
B4-4a-1201      ...................Y.....C.T..............
B4-4b-1201      ...................Y.....C.T..............
B4-5a-1201      ...................Y.....C.T..............
B4-5b-1201      ...................Y...N..................
B2-16a-1201     ...................Y.....C................
B2-16b-2101     ...................Y.....C................
B2-17b-1201     ...................Y.....C.............C..
B2-18b-1201     ...................Y.....C.............C..
B2-19b-1201     ...................Y...R........I.........
B2-37b-1201     ................................I.........
B4-39a-1201     ...................Y............I.........
B4-39b-1201     ...................Y............I.........
B2-41a-1201     ...................Y......................
B2-41b-1201     ...................Y......................
B2-42a-1201     ...................Y......................
B2-42b-1201     ...................Y......................
B2-43a-1201     ...................Y............T.........
B2-43b-1201     ...................Y......................
B2-60a-1201     ................T........C.............C..
B2-60b-1201     ................T........C.............C..
B2-61a-1201     .........................C.............C..
B2-61b-1201     ................T........C.............C..
B4-70a-1201     ...................Y......................
B4-70b-1201     ...................Y......................
```

Highlighted: mixture WT/mutant at 50%

```
                              10        20        30        40        50        60
                     ....|....|....|....|....|....|....|....|....|....|....|....|
Consensus C S-gene   MENTTSGFLGPLLVLQAGFFLLTRILTIPQSLDSWWTSLNFLGGAPTCPGQNSQSPTSNH
C2-8a-1201           ...........................M................................
C2-8b-1201           ............................................................
C1-11a-1201          .................V.......H..L.N.........................I...
C1-11b-1201          ..S.........................................................
C1-12b-1201          ..S..........G...V..........................................
C1-27b-1201          ..S.................................................L.......
C1-31a-1201          ..S.................................................L.......
C1-31b-1201          ..S.................................................L.......
C1-33a-1201          ..S....................K............................L.......
C1-33b-1201          ..S....................K............................L.......
C2-50a-1201          ............................................................
C2-50b-1201          ............................................................
C2-51a-1201          T...........H......S........................................
C2-51b-1201          T...........H......S........................................
C2-52a-1201          ..S....................................S...E.LK.............
C2-52b-1201          ..S....................................S...E.LK.............
C2-53a-1201          ..S....................................S...E.LK.............
C2-53b-1201          ..S....................................S...E.LK.............
C2-54a-1201          ............................................................
C2-54b-1201          ............................................................
C2-55a-1201          ............................................................
C2-55b-1201          ............................................................
C2-56a-1201          ............................................................
C2-56b-1156          ............................................................
C2-57a-1201          ............................................................
C2-57b-1201          ............................................................
C2-58b-1201          ..............................................A.............
C2-59b-1201          ............................................................
C2-62a-1201          ............................................................
C2-62b-1156          ............................................................
C1-63a-1201          .......................K............................L..L....
C2-64a-1201          ...........................................ET...............
C2-64b-1201          ...........................................ET...............

                              70        80        90       100       110       120
                     ....|....|....|....|....|....|....|....|....|....|....|....|
Consensus C S-gene   SPTSCPPICPGYRWMCLRRFIIFLFILLLCLIFLLVLLDYQGMLPVCPLLPGTSTTSTGP
C2-8a-1201           ............................................................
C2-8b-1201           ............................................................
C1-11a-1201          ..................H..................V......................
C1-11b-1201          ...............................T............................
C1-12b-1201          ...............................T............................
C1-27b-1201          ............................................................
C1-31a-1201          ........*...................................................
C1-31b-1201          *.......................................R...............R...
C1-33a-1201          ...............................T...G....K.............N.....
C1-33b-1201          ...............................T.S......K.............N.....
C2-50a-1201          ..................................W.........................
C2-50b-1201          ..................................W.........................
C2-51a-1201          .............L..R..................A.V..............STN...LS
C2-51b-1201          .............L..R..................A.V..............STN...LS
C2-52a-1201          ...C...T....................................................
C2-52b-1201          ...C...T....................................................
C2-53a-1201          ...C...T....................................................
C2-53b-1201          ...C...T....................................................
C2-54a-1201          ..................................W.........................
C2-54b-1201          ..................................W.........................
C2-55a-1201          ..................................W.........................
C2-55b-1201          ..................................W.........................
```

```
C2-56a-1201          .L................................W.........................
C2-56b-1156          .L................................W.........................
C2-57a-1201          ............................................................
C2-57b-1201          ............................................................
C2-58b-1201          ............................................................
C2-59b-1201          .......................F....................................
C2-62a-1201          ..................................W.........................
C2-62b-1156          .L..........................................................
C1-63a-1201          .........................................................A..
C2-64a-1201          ...................S....C......T............................
C2-64b-1201          ...................S....C......T............................

                              130       140       150       160       170       180
                     ....|....|....|....|....|....|....|....|....|....|....|....|
Consensus C S-gene   CKTCTIPAQGTSMFPSCCCTKPSDGNCTCIPIPSSWAFARFLWEWASVRFSWLSLLVPFV
C2-8a-1201           .....V......................................................
C2-8b-1201           .....V......................................................
C1-11a-1201          ............................................................
C1-11b-1201          .....M......................................................
C1-12b-1201          ............................................................
C1-27b-1201          .....T..................................................A...
C1-31a-1201          ........................R...............................A...
C1-31b-1201          ........................................................A...
C1-33a-1201          .....S......................................................
C1-33b-1201          .....S......................................................
C2-50a-1201          .....S......................................................
C2-50b-1201          .....S......................................................
C2-51a-1201          .......................................K....................
C2-51b-1201          .......................................K....................
C2-52a-1201          ............................................................
C2-52b-1201          ............................................................
C2-53a-1201          ............................................................
C2-53b-1201          ............................................................
C2-54a-1201          .....S......................................................
C2-54b-1201          .....S......................................................
C2-55a-1201          .....S......................................................
C2-55b-1201          .....S......................................................
C2-56a-1201          .....S......................................................
C2-56b-1156          .....S......................................................
C2-57a-1201          ............................................................
C2-57b-1201          ............................................................
C2-58b-1201          .....T......................................................
C2-59b-1201          ............................................................
C2-62a-1201          .....S......................................................
C2-62b-1156          ............................................................
C1-63a-1201          .....T......................................................
C2-64a-1201          ............................................................
C2-64b-1201          ............................................................
```

```
                         190       200       210       220
                   ....|....|....|....|....|....|....|....|....|..
Consensus C S-gene QWFVGLSPTVWLSVIWMMWYWGPSLYNILSPFLPLLPIFFCLWVYI*
C2-8a-1201         ..........................................
C2-8b-1201         ..........................................
C1-11a-1201        .......................N.....T............
C1-11b-1201        .....................A...H...NH....F......
C1-12b-1201        .....................A...H...NH....F......
C1-27b-1201        .............................N............
C1-31a-1201        .............................N............
C1-31b-1201        .............................N............
C1-33a-1201        .............................N............
C1-33b-1201        .............................N............
C2-50a-1201        ..........................................
C2-50b-1201        ..........................................
C2-51a-1201        ..............T..........N.......L......Y.
C2-51b-1201        ..............T..........N.......L......Y.
C2-52a-1201        ...A...............L............T.........
C2-52b-1201        ...A...............L............T.........
C2-53a-1201        ...A...............L............T.........
C2-53b-1201        ...A...............L...........CT.........
C2-54a-1201        ..........................................
C2-54b-1201        ..........................................
C2-55a-1201        ..........................................
C2-55b-1201        ..........................................
C2-56a-1201        ..........................................
C2-56b-1156        ..........................................
C2-57a-1201        ...A......................................
C2-57b-1201        ...A......................................
C2-58b-1201        .............A.....F......................
C2-59b-1201        .............A.....F......................
C2-62a-1201        ..........................................
C2-62b-1156        ..........................................
C1-63a-1201        .....................E.....T.N............
C2-64a-1201        .......................R....W....H........
C2-64b-1201        .......................R....W....H........
```
